# Slow ion concentration oscillations and multiple states in neuron–glia interaction—insights gained from reduced mathematical models

**DOI:** 10.3389/fnetp.2023.1189118

**Published:** 2023-05-22

**Authors:** Leiv Øyehaug

**Affiliations:** Faculty of Technology, Arts and Design, Oslo Metropolitan University, Oslo, Norway

**Keywords:** neuron–glia interplay, mathematical model, bursting, ion concentration dynamics, bifurcation analysis

## Abstract

When potassium in the extracellular space separating neurons and glia reaches sufficient levels, neurons may fire spontaneous action potentials or even become inactivated due to membrane depolarisation, which, in turn, may lead to increased extracellular potassium levels. Under certain circumstances, this chain of events may trigger periodic bursts of neuronal activity. In the present study, reduced neuron–glia models are applied to explore the relationship between bursting behaviour and ion concentration dynamics. These reduced models are built based on a previously developed neuron–glia model, in which channel-mediated neuronal sodium and potassium currents are replaced by a function of neuronal sodium and extracellular potassium concentrations. Simulated dynamics of the resulting two reduced models display features that are qualitatively similar to those of the existing neuron–glia model. Bifurcation analyses of the reduced models show rich and interesting dynamics that include the existence of Hopf bifurcations between which the models exhibit slow ion concentration oscillations for a wide range of parameter values. The study demonstrates that even very simple models can provide insights of possible relevance to complex phenomena.

## 1 Introduction

Neurons are the main carriers of signals and information in the brain. When a neuron fires an action potential, K^+^ ions are discharged from and Na^+^ ions are taken up by the neuron, causing elevated levels of K^+^ in the extracellular space (ECS) and of Na^+^ in neurons. This affects neuronal excitability and thus ion flux magnitudes, which may further enhance ECS K^+^ levels (Frankenhaeuser and Hodgkin, 1956; Fertziger and Ranck, 1970; Zuckermann and Glaser, 1970; Kager et al., 2000; Kager et al., 2007). During high action potential frequency, K^+^ accumulates in the ECS and excess ECS K^+^ is absorbed by surrounding glial tissue (Lux et al., 1986; Somjen, 2004), which, at low neuronal activity could have been removed by the neuronal sodium–potassium pump. Glia, which are at least as abundant as neurons in the brain, modulate neurons by K^+^ uptake, e.g., through the 3Na^+^/2K^+^ ATP-driven sodium–potassium pump, the inwardly rectifying K^+^ channel Kir4.1, the Na^+^/K^+^/2Cl^–^cotransporter (NKCC1) (see Østby et al. (2009) for references), and through potassium spatial buffering (Orkand et al., 1966; Halnes et al., 2013; Witthoft et al., 2013). Alterations in corresponding glial transmembrane ion fluxes will affect the dynamics of ${\left[K^{+}\right]}_{o}$ , as already explored by modelling (Øyehaug et al., 2012). Glial cells also contribute to regulation of cerebral blood flow by transport of K^+^ ions through glia from perisynaptic to perivascular ECS (Paulson and Newman, 1987; Farr and David, 2011).

With the increased recognition of glial cells as highly significant modulators of neuronal activity, the past decades have witnessed a growing interest in mathematical neuron–glia models (reviewed by Volman et al., 2012). Many of these models are characterised by highly detailed neuron models and glial models of much lower biophysical detail (Kager et al., 2000; Kager et al., 2002; Kager et al., 2007; Fröhlich et al., 2006; Cressman et al., 2009; Florence et al., 2009; Ullah and Schiff, 2010), such that the models can assess qualitative effects of glial potassium buffering but cannot provide insights into how individual glial actors operate during potassium clearance. Recent scientific and technological advances have stimulated the emergence of more complex neuron–glia models (e.g., Sibille et al., 2015; Du et al., 2016; Depannemaecker et al., 2022; Du et al., 2022; Liu et al., 2023), which are capable of reproducing and predicting quantitatively a wide range of phenomena. However, their complexity makes it challenging to pinpoint which part of the model is responsible for a certain type of behaviour. This is easier to do with low-complexity models.

Classical neuroscience models derived from the Hodgkin and Huxley formalism (e.g., Hodgkin and Huxley, 1952) mostly neglect the variation in ion concentrations due to the short time scales of action potential dynamics. On longer time scales, ion concentration variability and its effect on neuronal excitability cannot be neglected. Mathematical neuron–glia models are thus generally composed of a fast neuron model and a slow glia model, in which the latter typically describes ion concentration dynamics in which time is measured in seconds. Therefore, when studying phenomena on this time scale, it may be beneficial to create models that facilitate the analysis of the slow ion concentration dynamics by developing alternative representations of the fast time scale dynamics (e.g., Cressman et al., 2009). Derivation of such reduced models and application of these models to describe and explain phenomena that are impacted by the neuron–glia interaction represent one of the outputs of the present study.

Previously, a combination of a glia model (Østby et al., 2009) with the neuron–ECS model of Kager et al. (2000) was developed and applied (Øyehaug et al., 2012) to investigate ECS K^+^ $\left({\left[K^{+}\right]}_{o}\right)$ dynamics and its effect on neuronal excitability, and to examine how glial membrane processes modulate neuronal excitability through the action of ${\left[K^{+}\right]}_{o}$ . Model simulations of that study revealed that spontaneous bursts of neuronal activity and oscillations in ion concentrations were generated in certain glial parameter regimes, a discovery made independent of the phenomenon having been reported in several experiments (McBain, 1994; Jensen and Yaari, 1997; Feng and Durand, 2006; Ziburkus et al., 2006) and in numerous theoretical modelling studies (Bazhenov et al., 2004; Fröhlich et al., 2006; Cressman et al., 2009; Barreto and Cressman, 2011). The present paper extends the work from Øyehaug et al. (2012) by developing reduced neuron–glia models to disclose mechanisms that underlie the bursting behaviour. Since these models do not have action potentials, examining how bursts emerge is equivalent to examining how ion concentration oscillations emerge in the low-complexity reduced model. Developing the reduced models, a function of [K^+^]_o_ and the neuronal Na^+^ concentration $\left({\left[Na^{+}\right]}_{n}\right)$ is fitted to neuronal ion fluxes obtained in simulations using the model of Kager et al. (2000). The resulting reduced models are investigated using simulation and bifurcation analysis. Although the study, to some extent, replicates the modelling, methods, and results of Cressman et al. (2009) and Barreto and Cressman (2011), the more biophysically detailed glia model in the present study compared to these studies allows the assessment of the role of various glial actors in the neuron–glia interaction.

## 2 Materials and methods

**FIGURE 1:**
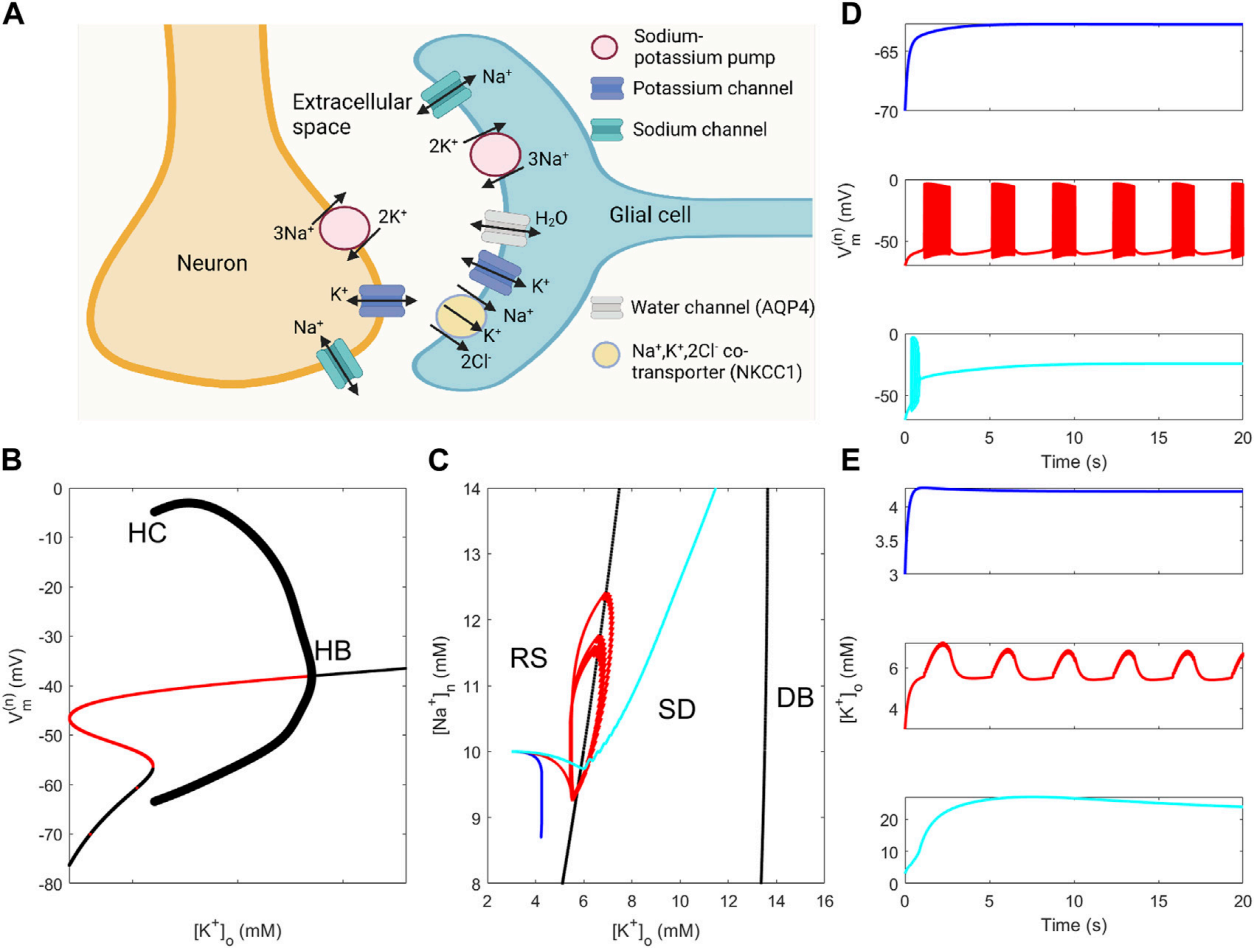
Properties of the neuron–ECS–glia system and of neuron and neuron–glia models. **(A)** Schematic representation of the neuron–glia system with channels, pumps, and cotransporters in the neuron and glia membranes (depicted sodium and potassium channels in the neuron membrane comprise several channels). **(B)** Bifurcation diagram of the model of Kager et al. (2000) using [K^+^]_o_ as the bifurcation parameter [(Na^+^)_n_ is fixed at 10 mM, and model parameter values are given in Supplementary Material]. “HB” and “HC” indicate locations of a Hopf bifurcation and a heteroclinic bifurcation, respectively. **(C)** Two-parameter bifurcation diagram of the model of Kager et al. (2000) using [K^+^]_o_ and [Na^+^]_n_ as bifurcation parameters showing how curves that correspond to the HB and HC bifurcations in **(B)** separate the parameter plane into regions corresponding to the resting state (RS), spontaneous discharge (SD), and depolarisation block (DB) behaviour. Curves displayed are solution orbits corresponding to the dynamics displayed in **(D)** and **(E)** using the same colour coding. **(D)**–**(E)**: Dynamics of neuronal membrane potential **(D)** and of [K^+^]_o_ **(E)** obtained from simulations using the full neuron–glia model (parameter values are given in Supplementary Material). The glial sodium–potassium pump rate $J_{\text{NaKATPase,max}}^{\left(\text{g}\right)}$ is multiplied by factors of 0.8 (top panels in D and E), 0.65 (middle), and 0.55 (bottom) to generate different types of behaviour.

The system under study comprises the neuron, the glial cell surrounding the neuron, and the ECS separating the two cells. This *neuron–ECS–glia* system (also sometimes referred to as the *tripartite synapse*) is depicted with its actors in the neuronal and glial membranes in Figure 1A. In the scenario in which an active neuron increases its own activity by releasing large quantities of K^+^ into the ECS, potentially causing harmful seizure activity, the key question of the present study is to which extent the sodium–potassium pump and the NKCC1 cotransporter in the glial membrane can clear ECS K^+^ and thereby contribute to maintaining a sound microenvironment in the surroundings of the neuron. This question is addressed using the reduced neuron–glia models that are derived from the neuron–glia model developed by Øyehaug et al. (2012).

### 2.1 The neuron–glia model

The neuron–glia model is an ordinary differential equation (ODE) model that describes ion and water transport through the membranes separating the neuron, the ECS, and the glial cell. The model is, crudely speaking, composed of the glia model developed by Østby et al. (2009) and the neuron model by Kager et al. (2000). Examples of simulated neuronal membrane potential and [K^+^]_o_ dynamics using the neuron–glia model defined by Eqs 1a–6 are shown in Figures 1D, E. The model exhibits resting state (RS) behaviour when the glial sodium–potassium pump rate is slightly reduced (top panels), shows behaviour alternating between RS and spontaneous discharges (SDs), i.e., *spontaneous bursting* (SB), when the pump rate is moderately reduced (middle panels), and depolarisation block (DB) behaviour when the pump rate is severely reduced (bottom panels). In the DB case, the neuron membrane is highly depolarised and [K^+^]_o_ shows extreme levels. RS, SB, and DB represent the three modes of behaviour in the dynamical repertoire of the neuron–glia model.

To assess the significance of ion concentration dynamics on mode selection, the neuron model was subjected to bifurcation analysis with [K^+^]_o_ and [Na^+^]_n_ as bifurcation parameters. Fixing [Na^+^]_n_ to 10 mM, the neuron model exhibits RS behaviour for low-to-moderate [K^+^]_o_ levels, SD behaviour for [K^+^]_o_ approximately in the range 6–13 mM, and DB behaviour for [K^+^]_o_ beyond 13 mM (Figure 1B). Extending the bifurcation analysis to allow variation also in [Na^+^]_n_ resulted in a two-dimensional bifurcation plot where the locations of the Hopf and heteroclinic bifurcations are tracked throughout the $\left({\left[K^{+}\right]}_{o},{\left[Na^{+}\right]}_{n}\right)$ -plane. The two-parameter analysis separates the plane into three regions corresponding to the three dynamics modes (Figure 1C). The solution orbits corresponding to the dynamics in Figures 1D, E are depicted in the diagram demonstrating RS dynamics (black curve), SD and bursting behaviour (red), and DB behaviour (cyan). The red curve repeatedly enters and exits the SD region (Figure 1C), indicative of the bursting behaviour (SB) observed in Figures 1D, E, middle panel.

The neuron dynamics is described by a modified version of the neuron model of Kager et al. (2000) that describes the membrane potential dynamics that result from K^+^ and Na^+^ transmembrane currents and the kinetics of channel gates. The associated ODEs of the neuron model read

$$C_{\text{m}}\frac{dV_{\text{m}}^{\left(n\right)}}{dt}=10^{-3}\left(-I_{\text{Na}}^{\left(n\right)}I_{\text{K}}^{\left(n\right)}-I_{\text{leak,f}}-I_{\text{NaKATPase,n}}\right),$$ (1a)

$$dx/dt=\alpha_{x}\left(1-x\right)-\beta_{x}x,$$ (1b)

where *C*_*m*_ = 1 *μF* cm^−2^ is the neuron membrane specific capacitance, $-I_{\text{Na}}^{\left(n\right)}=-I_{\text{Na,T}}-I_{\text{Na,P}}-I_{\text{leak,Na}}$ represent transient, persistent, and leak sodium currents, respectively, and $-I_{\text{K}}^{\left(n\right)}=-I_{\text{K,DR}}-I_{\text{K,A}}-I_{\text{leak,K}}$ represent delayed rectifier, transient, and leak potassium currents, respectively. Furthermore, *x* is any of the activating or inactivating gates. Exact expressions for the currents, forward and backward rates *α*_*x*_ and *β*_*x*_, and parameter values of the neuron model can be found in Øyehaug et al. (2012) and in Supplementary Material.

The glia model describes the time rate of change of the number of ions S (denoted *N*_S,g_ for the number of ions within glia or *N*_S,o_ in the ECS, where S can be sodium, potassium, and chloride), the time rate of change of the glial volume by ODEs, and the change of the glial membrane potential as a function of time by an algebraic equation. The number of each ion species S (Na^+^, K^+^, and Cl^−^) per unit of glial area in the glia compartment is given by the product *N*_S,j_ = *w*_j_[S]_j_, where j is either g or o and *w*_g_ represents the ratio between the glial cell volume and glial membrane area. Provided the ECS and glial volumes are modelled by glial membrane processes, a precise geometrical specification of the region of interest is not required (Chen and Nicholson, 2000; Østby et al., 2009); it suffices to specify the ratio between the ECS volume and the associated glial volume and the ratio between the glial membrane area and glial volume. The model describes sodium, potassium, and chloride channels, as well as the sodium–potassium pump, the NKCC1 cotransporter, and water channels in the glial membrane (depicted in Figure 1A). Compared to the model used by Øyehaug et al. (2012), the model implemented in this study neglects bicarbonate ions and the electrogenic sodium bicarbonate cotransporter (NBC).

The neuron–glia model (hereafter referred to as the *full model*) is composed of the aforementioned Eqs 1a, 1b and the following ODEs for the number of ions in the neuronal, ECS, and glia compartments;

$$\frac{dN_{K^{+},o}}{dt}=\Phi_{\text{S}}\left[J_{\text{Na}}^{\left(n\right)}-3J_{\text{NaKATPase}}^{\left(n\right)}-\left(J_{\text{K}}^{\left(g\right)}+2J_{\text{NaKATPase}}^{\left(g\right)}+J_{\text{NKCC}1}\right)\right],$$ (2a)

$$\frac{dN_{Na^{+},n}}{dt}=\Phi_{\text{S}}\left[J_{\text{Na}}^{\left(n\right)}-3J_{\text{NaKATPase}}^{\left(n\right)}\right],$$ (2b)

$$\frac{dN_{Na^{+},g}}{dt}=\Phi_{\text{S}}\left[J_{\text{Na}}^{\left(g\right)}-3J_{\text{NaKATPase}}^{\left(g\right)}+J_{\text{NKCC}1}\right],$$ (2c)

$$\frac{dw_{\text{g}}}{dt}=\Phi_{\text{w}}L_{p}\left[\Pi_{\text{g}}-\Pi_{\text{o}}\right],$$ (2d)

where $\Pi_{\text{g}}={\left[Na^{+}\right]}_{g}+{\left[K^{+}\right]}_{g}+{\left[Cl^{-}\right]}_{g}+X_{\text{g}}/w_{\text{g}}$ and $\Pi_{\text{o}}={\left[Na^{+}\right]}_{o}-{\left[K^{+}\right]}_{o}-{\left[Cl^{-}\right]}_{o}$ are the glial and ECS osmolarities, respectively, $J_{\text{NaKATPase}}^{\left(g\right)}$ and $J_{\text{NaKATPase}}^{\left(n\right)}$ are the glial and neuronal sodium–potassium pump fluxes (pump rates), respectively, *J*_NKCC1_ is the electrochemically induced ion flux mediated by the NKCC1 cotransporter, and Φ_S_ = 10^–2^ and Φ_w_ = 10 are conversion factors that ensure matching units in the ODEs (explained in Supplementary Material). Exact expressions for the fluxes are given in Table 1. *X*_g_ is the number of negatively charged impermeable ions trapped within the glial cell divided by the glial cell area *A*. All ion species are subject to ion number conservation, i.e.,

$$N_{\text{S}}^{\left(n\right)}+N_{\text{S}}^{\left(o\right)}+N_{\text{S}}^{\left(g\right)}=N_{\text{S}},\;S=K^{+},\;Na^{+},\;Cl^{-},$$ (3)

where *N*_S_ is constant. Furthermore, the sum of the ECS and glia volumes and the neuron volume are assumed constant such that *w*_o_ + *w*_g_ = *w*_tot_ and *w*_n_ are constants. The fact that the expression $J_{\text{Na}}^{\left(n\right)}-3J_{\text{NaKATPase}}^{\left(n\right)}$ occurs in Eq. 2a and in Eq. 2b is due to the requirement of charge electroneutrality and is detailed below. The details of the rewriting of the present model equations from the model equations in Øyehaug et al. (2012) are given in Supplementary Material.

**TABLE 1:** Ion flux densities in the glia model and the sodium–potassium pump ion flux density in the neuron model. The glial Nernst potential of ion species S is $E_{\text{S}}^{\left(g\right)}=\left(\Psi /z_{\text{S}}\right)\ln\left({\left[S\right]}_{o}/{\left[S\right]}_{g}\right)$ , where *z*_S_ is the valence of S and *Ψ* = *RT*/*F* ≈25.8 mV, where *R*, *T*, and *F* are the gas constant, temperature, and Faraday constant, respectively. The ion flux through NKCC1 is modelled in a Nernst-like fashion (Lauf and Adragna, 2000; Dronne et al., 2006; Dronne et al., 2007). All concentrations are given in mM.

Term	Expression	Description
$J_{\text{Na}}^{\left(g\right)}$	$-\frac{g_{\text{Na}}}{F}\left(V_{\text{m}}^{\left(g\right)}-E_{\text{Na}}^{\left(g\right)}\right)$	Glial sodium channel flux density
$J_{\text{K}}^{\left(g\right)}$	$-\frac{g_{\text{K}}}{F}\left(V_{\text{m}}^{\left(g\right)}-E_{\text{K}}^{\left(g\right)}\right)$	Glial potassium channel flux density
*J*_NKCC1_	$\frac{g_{\text{NKCC}1}}{F}\Psi \ln\left(\frac{{\left[Na^{+}\right]}_{o}}{{\left[Na^{+}\right]}_{g}}\frac{{\left[K^{+}\right]}_{o}}{{\left[K^{+}\right]}_{g}}{\left(\frac{{\left[{Cl}^{-}\right]}_{\text{o}}}{{\left[Cl^{-}\right]}_{g}}\right)}^{2}\right)$	Glial NKCC1 ion flux density
$J_{\text{NaKATPase}}^{\left(g\right)}$	$J_{\text{NaKATPase,max}}^{\left(g\right)}\frac{{\left[Na^{+}\right]}_{g}^{1.5}}{{\left[Na^{+}\right]}_{g}^{1.5}+K_{\text{m,Na}}^{1.5}}\frac{{\left[K^{+}\right]}_{o}}{{\left[K^{+}\right]}_{o}+K_{\text{m,K}}}$	Glial sodium–potassium pump ion flux density
$J_{\text{NaKATPase}}^{\left(n\right)}$	$J_{\text{NaKATPase,max}}^{\left(n\right)}\frac{{\left[Na^{+}\right]}_{n}^{1.5}}{{\left[Na^{+}\right]}_{n}^{1.5}+K_{\text{m,Na}}^{1.5}}\frac{{\left[K^{+}\right]}_{o}}{{\left[K^{+}\right]}_{o}+K_{\text{m,K}}}$	Neuronal sodium–potassium pump ion flux density

### 2.2 Electroneutrality

The requirement of charge neutrality (Johnston and Wu, 1994) is enforced within each compartment. Glial electroneutrality is ensured by assuming the glial chloride concentration to be given by the following equation:

$${\left[{Cl}^{-}\right]}_{\text{g}}={\left[{Na}^{+}\right]}_{\text{g}}+{\left[K^{+}\right]}_{\text{g}}-\rho \frac{X_{\text{g}}}{w_{\text{g}}}.$$ (4)

Here, *ρ* is the average charge of the negatively charged impermeable ions relative to the elementary charge. Multiplication of Eq. 4 by *w*_*g*_ followed by differentiation gives an algebraic equation that translates to the assumption that the total glial transmembrane electric current is at every instant zero, giving for the glial membrane potential $V_{\text{m}}^{\left(g\right)}$ ;

$$V_{\text{m}}^{\left(g\right)}=\frac{g_{\text{Na}}E_{\text{Na}}^{\left(g\right)}+g_{\text{K}}E_{\text{K}}^{\left(g\right)}+g_{\text{Cl}}E_{\text{Cl}}^{\left(g\right)}-J_{\text{NAKATPase}}^{\left(g\right)}F}{g_{\text{Na}}+g_{\text{K}}+g_{\text{Cl}}},$$ (5)

where the glial Nernst potentials of the ion species S ( $E_{\text{S}}^{\left(g\right)}$ , S = Na^+^, K^+^, and Cl^−^) and the glial sodium–potassium pump rate $J_{\text{NAKATPase}}^{\left(g\right)}$ are defined in the caption to Table 1.

Since the total glial transmembrane current is zero and the total neuronal transmembrane current is generally non-zero, the additional assumption that the neuronal chloride flux can be neglected is imposed to ensure ECS electroneutrality. Then, the ECS chloride level is

$${\left[{Cl}^{-}\right]}_{\text{o}}={\left[Na^{+}\right]}_{\text{o}}+{\left[K^{+}\right]}_{\text{o}}.$$ (6)

Finally, neglecting Cl^−^neuronal currents and assuming that neuronal K^+^ and Na^+^ currents are equal in magnitude but oppositely directed (which explains the identical terms in Eqs 2a, 2b) together ensure that the neuron is electroneutral.

### 2.3 Model reduction

The model reduction process that leads to the creation of the two reduced models involves i) replacing neuronal ion fluxes at given [Na^+^]_n_ and [K^+^]_o_ by the expression obtained when fitting a suitable function to the ion fluxes computed using the neuron model and ii) assuming that the sum of ECS K^+^ and Na^+^ levels is constant. Step i) is used to create the first reduced model (RM1). When additionally invoking step ii), the second reduced model (RM2) is derived.

#### 2.3.1 Approximation of neuronal currents

In the first step of the model reduction process, the neuronal transmembrane sodium flux is replaced by a function of [K^+^]_o_ and ${\left[Na^{+}\right]}_{n}$ as follows: The neuron model ODEs (Kager et al., 2000) are numerically solved on a representative domain in the $\left({\left[K^{+}\right]}_{o}{\left[Na^{+}\right]}_{n}\right)$ plane, such that all modes of dynamics (RS, SD, and DB) are covered for a range of pairs $\left({\left[K^{+}\right]}_{o}{\left[Na^{+}\right]}_{n}\right)$ , followed by estimation of the voltage-gated channel-mediated sodium flux density. The function *J* to be fitted to the simulation data is defined as follows:

$$J\left({\left[K^{+}\right]}_{o},{\left[Na^{+}\right]}_{n}\right)=y_{1}+y_{2}F\left(u\right)\left(1-G\left(u\right)\right)+y_{3}\exp\left[-\frac{{\left[Na^{+}\right]}_{n}}{y_{4}}\right]G\left(u\right),$$ (7a)

$$F\left(u\right)=\frac{y_{5}-u}{1+\exp\left[Mu\right]},\;u={\left[Na^{+}\right]}_{n}-y_{6}{\left[K^{+}\right]}_{o}+y_{7},$$ (7b)

$$G\left(v\right)=\frac{1}{1+\exp\left[Mv\right]},\;v={\left[Na^{+}\right]}_{n}-y_{8}{\left[K^{+}\right]}_{o}+y_{9}.$$ (7c)

**FIGURE 2:**
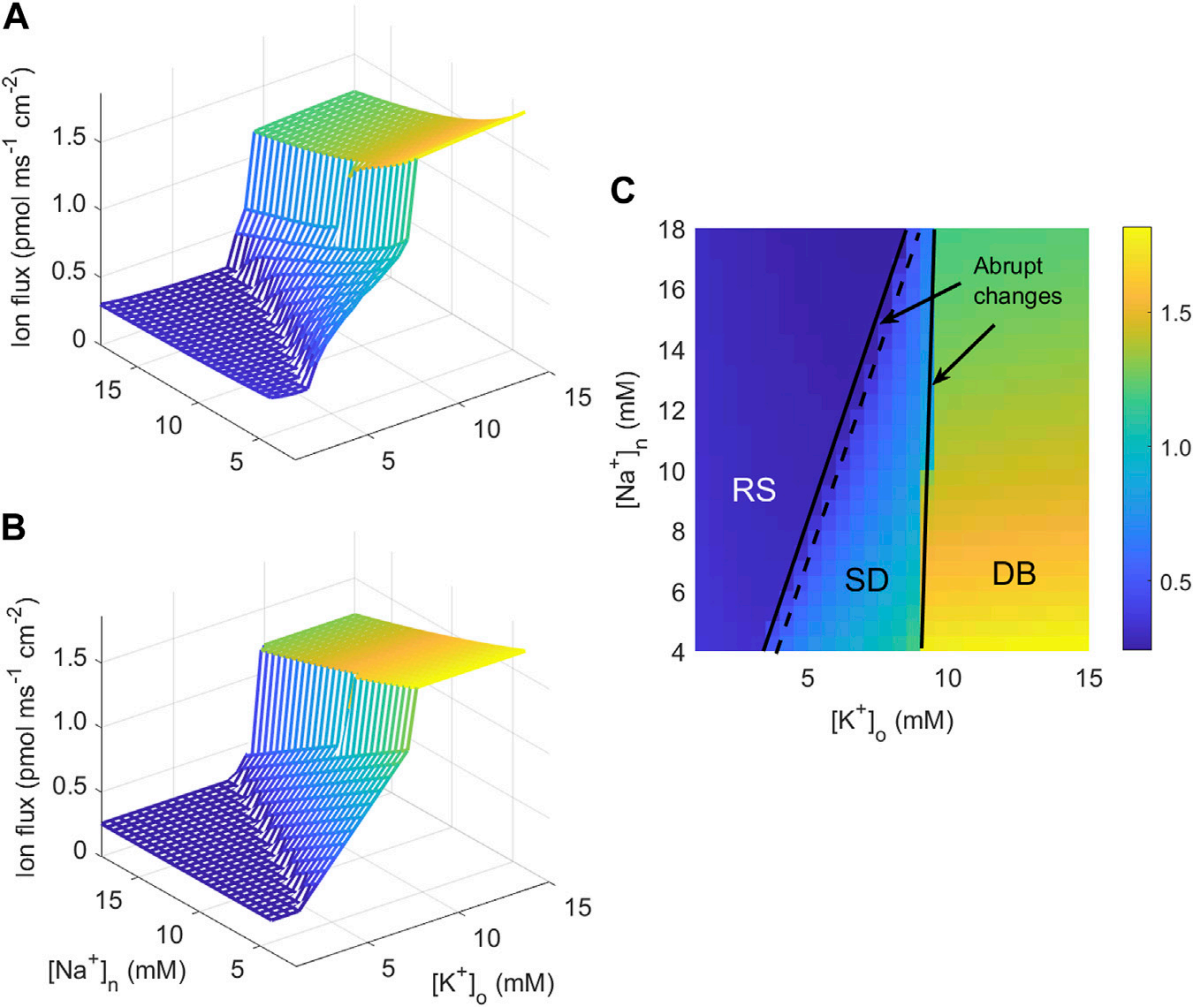
Calculated and fitted sodium transmembrane neuronal current. **(A)** Magnitude of the sodium current into the neuron as a function of [K^+^]_o_ and [Na^+^]_n_ computed from the neuronal model of Kager et al. (2000). **(B)** Best fit to the current plotted in **(A)** to the function given in Eq. 7a obtained by the parameters *y*_1_ = 0.367, *y*_2_ = 25.52, *y*_3_ = 6.39, *y*_4_ = 35.38, *y*_5_ = 1.10, *y*_6_ = 2.51, *y*_7_ = 3.33, *y*_8_ = 16.38, and *y*_9_ = 137.9 (the unit for *y*_1_ and *y*_3_ is pmol ms^−1^ cm^−2^, for *y*_2_: pmol ms^−1^ cm^−2^ mM^−1^, for *y*_4_, *y*_5_, *y*_7_, and *y*_9_: mM. *y*_6_ and *y*_8_ are unit-free). **(C)** Contour plot of the current in **(A)** as a function of [K^+^]_o_ and [Na^+^]_n_ with indications of the expected behaviour in three regions of the ([K^+^]_o_,[Na^+^]_n_)-plane.

The parameter *M* is set to 50 such that *F* and *G* are step-like in the vicinity of *u* = 0 and *v* = 0, respectively, *F* is approximately zero when $\left({\left[K^{+}\right]}_{o},{\left[Na^{+}\right]}_{n}\right)$ is located above the straight line defined by *u* = 0 and approximately equal to *y*_5_ − *u* when ([K^+^]_o_,[Na^+^]_n_) is below *u* = 0. Moreover, *G* is approximately zero when $\left({\left[K^{+}\right]}_{o},{\left[Na^{+}\right]}_{n}\right)$ is located above the straight line defined by *v* = 0 and approximately equal to 1 when $\left({\left[K^{+}\right]}_{o},{\left[Na^{+}\right]}_{n}\right)$ is below *v* = 0. This means that the current density is approximately equal to the constant level *y*_1_ in the region called “RS” in Figure 2C, approximately equal to the linear function *y*_1_ + *y*_2_(*y*_5_ − *u*) in “SD” and to $y_{1}+y_{3}\exp\left[-{\left[Na^{+}\right]}_{n}/y_{4}\right]$ , which is approximately constant, in “DB.” Mathematically, this means that

$$J\approx \left\{\begin{array}{cc} y_{1}\; & \text{in RS},\\ y_{1}+y_{2}\left(y_{5}-u\right)\; & \text{in SD},\\ y_{1}+y_{3}\exp\left[-{\left[Na^{+}\right]}_{n}/y_{4}\right]\; & \text{in DB}, \end{array}\right.$$ (8)

which is in agreement with the computed ion flux (Figures 2A, C). The function *J* is fitted to the simulation data by minimising a cost function defined as the sum of squared deviations between data and the function *J* using the MATLAB function fminsearch. The estimated parameter values are given in the caption to Figure 2.

#### 2.3.2 Development of reduced models

In order to derive the first reduced model RM1, the aforementioned fitting procedure was invoked to replace the neuronal channel-mediated sodium current *J*_Na_ in Eqs 2a, 2b by the function in Eqs 7a, 7c. Then, the ODEs Eqs 1a, 1b describing action potential dynamics are not needed, such that the model equations for RM1 are

$$\frac{dN_{K^{+},o}}{dt}=\Phi_{\text{S}}\left[J-3J_{\text{NaKATPase}}^{\left(n\right)}-\left(J_{\text{K}}^{\left(g\right)}-2J_{\text{NaKATPase}}^{\left(g\right)}+J_{\text{NKCC}1}\right)\right],$$ (9a)

$$\frac{dN_{Na^{+},n}}{dt}=\Phi_{\text{S}}\left[J-3J_{\text{NaKATPase}}^{\left(n\right)}\right],$$ (9b)

$$\frac{dN_{Na^{+},g}}{dt}=\Phi_{\text{S}}\left[J_{\text{Na}}^{\left(g\right)}-3J_{\text{NaKATPase}}^{\left(g\right)}+J_{\text{NKCC}1}\right],$$ (9c)

$$\frac{dw_{\text{g}}}{dt}=\Phi_{\text{w}}\;L_{p}\left[\Pi_{\text{g}}-\Pi_{\text{o}}\right].$$ (9d)

For a range of parameter values in the full model and in RM1, [K^+^]_o_ + [Na^+^]_o_ shows limited variation (Supplementary Figure S1). In the second step of the model reduction process, it is thus additionally assumed that *the sum of K* ^*+*^ *and Na* ^*+*^ *ECS concentrations,* [*K* ^*+*^]_*o*_ *+* [*Na* ^*+*^]_*o*_ *, is constant*. This allows the omission of the variable $N_{Na^{+},g}$ from the model and causes the ECS and glia models to be constant (proven in Supplementary Material). A further consequence is that the sum ${\left[Na^{+}\right]}_{\text{g}}+{\left[K^{+}\right]}_{\text{g}}$ is also constant, and due to glial and ECS electroneutrality, both ${\left[Cl^{-}\right]}_{\text{o}}$ and ${\left[Cl^{-}\right]}_{\text{g}}$ must be constant. Then, the second reduced model equations are obtained from RM1 Eqs 9a–9d by omitting the equations for $N_{Na^{+}}^{\left(g\right)}$ and *w*_g_;

$$\frac{dN_{K^{+},o}}{dt}=\Phi_{\text{S}}\left[J-3J_{\text{NaKATPase}}^{\left(n\right)}-\left(J_{\text{K}}^{\left(g\right)}-2J_{\text{NaKATPase}}^{\left(g\right)}+J_{\text{NKCC}1}\right)\right],$$ (10a)

$$\frac{dN_{Na^{+},n}}{dt}=\Phi_{\text{S}}\left[J-3J_{\text{NaKATPase}}^{\left(n\right)}\right].$$ (10b)

In RM2, ion number variables could have been replaced by ion concentrations but were kept to maintain consistency between all models.

## 3 Results

In Øyehaug et al. (2012), it was observed that the neuron–glia model exhibited slow ion concentration oscillations when the glial sodium–potassium pump rate was sufficiently weakened. In the present study, in addition to studying the effect of the pump rate, the dependence of slow oscillations on NKCC1-mediated ion uptake and on glial K^+^ levels will be examined in RM1 and RM2 using bifurcation analysis, phase plane analysis, and numerical simulations. Bifurcation analyses and numerical simulations were performed using XPPAUT software (Ermentrout, 2012) and the MATLAB ODE solver ode113, respectively.

### 3.1 Comparison of simulated model dynamics

**FIGURE 3:**
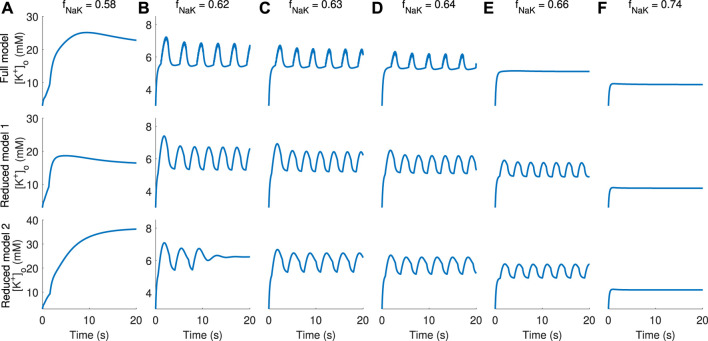
Comparison of [K^+^]_o_ dynamics obtained in simulations using the full and reduced models. Columns **(A–F)** display [K^+^]_o_ dynamics for increasing values of the sodium–potassium pump rate obtained by multiplication by a factor *f*_NaK_ in the range 0.58–0.74, indicated at the top of each column. Full model dynamics are displayed in the top panel, RM1 in the middle, and RM2 in the bottom panel.

To assess whether the full and reduced models show similar behaviour, model simulations were performed for a range of values for the glial sodium–potassium reduction factor *f*_NaK_, which is multiplied by the rate $J_{\text{NaKATPase}}^{\left(g\right)}$ in order to generate different modes of dynamics for [K^+^]_o_ (Figure 3). At sufficient reduction (*f*_NaK_ = 0.58, Figure 3A), all models display depolarisation block (DB) behaviour, where [K^+^]_o_ converges to extreme levels, especially in RM2. For less pronounced reductions [*f*_NaK_ in the range (0.62, 0.66), Figures 3B–E], slow oscillations are the rule, although for the largest value in this range (*f*_NaK_ = 0.66, Figure 3E), the full model displays RS behaviour in which RM1 and RM2 show oscillating solutions. Furthermore, when *f*_NaK_ = 0.62, the RM2 shows vanishing [K^+^]_o_ oscillations and convergence to a semi-elevated level (Figure 3B, bottom). This behaviour represents an anomaly and is discussed and explained by the following bifurcation analysis. All models predict the system to be in the resting state for moderate reduction of the sodium–potassium rate (*f*_NaK_ = 0.74, Figure 3F). Although the simulations do not demonstrate a precise quantitative agreement between the different models, all exhibit gradual changes of the dynamics from RS via SB to DB behaviour with decreasing pump rate.

### 3.2 Dependence on the glial sodium–potassium pump rate

**FIGURE 4:**
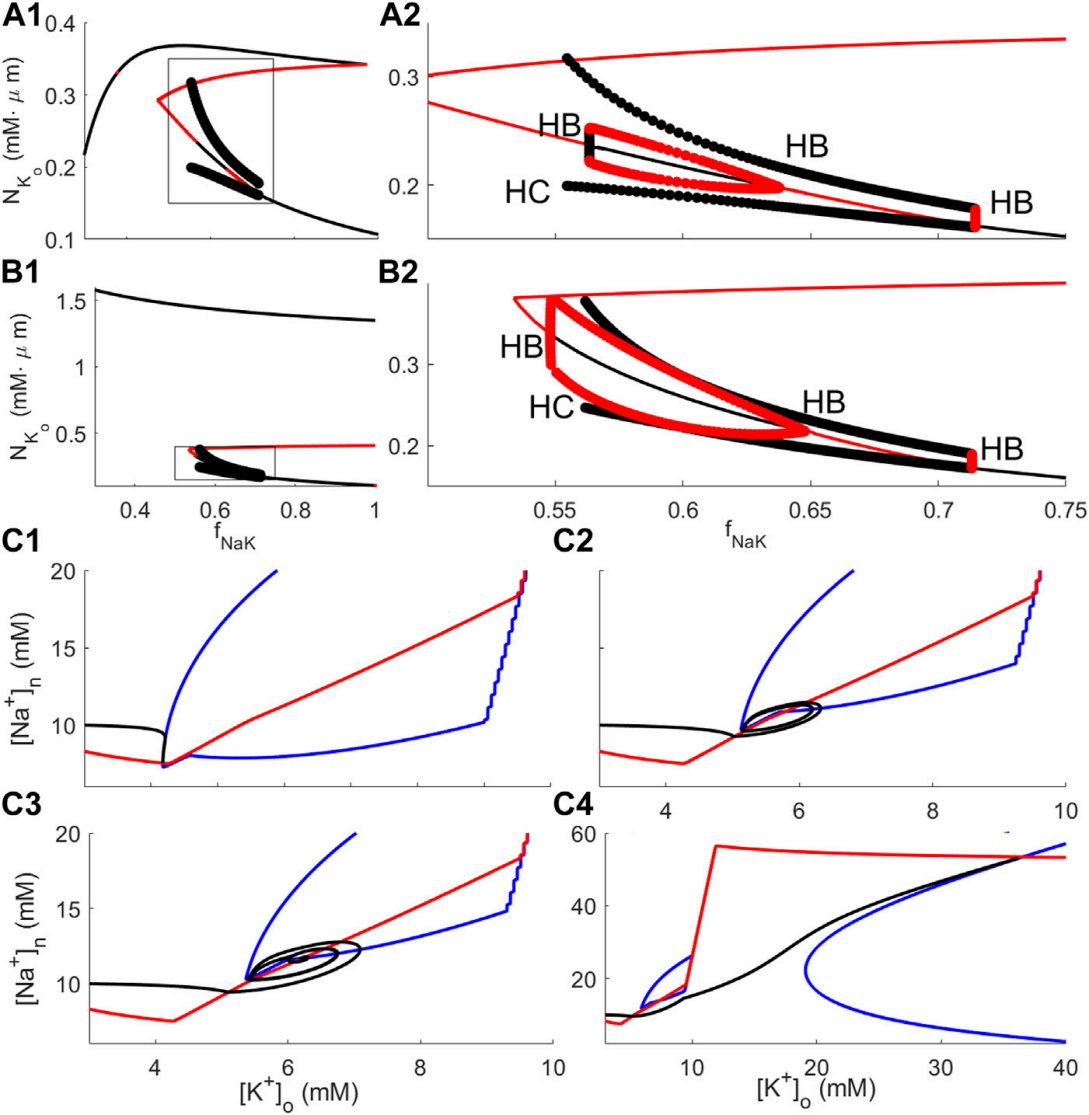
Bifurcation and phase plane analysis for RM1 and RM2. **(A1-A2)**: Bifurcation diagram of RM1 with the sodium–potassium pump rate reduction factor *f*_NaK_ as the bifurcation parameter. **(A1)** shows the full diagram, and **(A2)** shows details of the diagram within the rectangle depicted in **(A1)**. The black curve indicates stable steady states; red curve, unstable steady states; black filled circles, stable oscillatory solutions; and red filled circles, unstable oscillatory solutions. “HB” and “HC” indicate locations of Hopf and heteroclinic bifurcations, respectively. **(B1–B2)**: Same as **(A1–A2)** for RM2. **(C1–C4)**: Solution orbits and phase plane analysis for RM2 in the $\left({\left[K^{+}\right]}_{o},{\left[Na^{+}\right]}_{n}\right)$ -plane. In all plots, red and blue curves indicate nullclines of $dN_{{\text{Na}}^{+},\text{n}}/dt$ and $dN_{{\text{K}}^{+},\text{o}}/dt$ , respectively, and black curves are solution orbits. The depicted dynamics correspond to different values of *f*_NaK_; **(C1)** *f*_NaK_ = 0.74, **(C2)** *f*_NaK_ = 0.64, **(C3)** *f*_NaK_ = 0.62, and **(C4)** *f*_NaK_ = 0.58.

Spontaneous bursting was observed in the full model when the glial sodium–potassium pump rate was significantly reduced (Øyehaug et al., 2012); see also Figures 1C, D. In order to assess the propensity of the reduced models to show the same kind of behaviour, bifurcation analyses with the glial sodium–potassium pump rate reduction factor *f*_NaK_ as the bifurcation parameter were performed for RM1 and RM2. This analysis shows that both models possess a rich dynamical repertoire (Figures 4A1, A2, B1, B2).

#### 3.2.1 Bifurcation analysis of reduced models

The full bifurcation diagrams for RM1 and RM2 (Figures 4A1, B1, respectively) show the same qualitative behaviour; for modest pump rate reductions, i.e., for *f*_NaK_ near 1, [K^+^]_o_ is at moderate levels (corresponding to resting state–RS–solutions), and for smaller *f*_NaK_, there is a regime of values in which solutions are oscillating (spontaneous bursting–SB). For all values of *f*_NaK_, there is an elevated K^+^ state with very high K^+^ levels, especially in RM2 (depolarisation block–DB). Thus, the DB state coexists with the RS and with the SB state. Enlarging the areas within the rectangles in Figures 4A1, B1, further details of the bifurcation analysis for RM1 and RM2 are revealed (Figures 4A2, B2, respectively). The bifurcation diagrams of RM1 and RM2 are qualitatively similar; both show RS behaviour for *f*_NaK_ just below 1, SB behaviour in an intermediate range for *f*_NaK_, and no stable steady state below some threshold values for *f*_NaK_. The values which separate different modes of dynamical behaviour are *Hopf bifurcations* (indicated by “HB”) where, as stable steady states go unstable, stable limit cycle solutions emerge (or the opposite; as unstable steady states go stable, unstable limit cycle solutions emerge). Surprisingly, in both diagrams, in addition to the Hopf bifurcation separating the stable RS and stable bursting oscillations (approximately at *f*_NaK_ = 0.72 in both diagrams), there is one Hopf bifurcation where the unstable steady state that coexists with stable limit cycle solutions goes stable when *f*_NaK_ is decreasing and unstable limit cycle solutions emerge (roughly at *f*_NaK_ = 0.64 in both diagrams) and one where the stable steady state goes unstable again when *f*_NaK_ is decreasing (roughly at *f*_NaK_ = 0.54 in RM1 and at *f*_NaK_ = 0.56 in RM2). In this case, three stable states coexist, the DB state, the SB state, and the semi-elevated [K^+^]_o_ state, i.e., *tristability*. Figure 3B, bottom, shows [K^+^]_o_ dynamics using RM2 with *f*_NaK_ = 0.62, i.e. in the parameter regime of tristability. Solutions exhibit vanishing oscillations and subsequent convergence to a stable steady state, consistent with the bifurcation diagram at this value of *f*_NaK_ which predicts the existence of unstable oscillatory solutions in coexistence with a stable semi-elevated state for [K^+^]_o_. The tristability phenomenon will further be elaborated in the Discussion. Finally, the points where the periodic limit cycle solutions vanish (approximately at *f*_NaK_ = 0.55 in RM1 and at *f*_NaK_ = 0.56 in RM2) are *heteroclinic bifurcations* (indicated by “HC” in the diagrams).

#### 3.2.2 Phase plane analysis of RM2

The dependence of the RM2 dynamics on *f*_NaK_ was examined by solving the RM2 model Eqs 10a, 10b for *f*_NaK_ = 0.74, 0.64, 0.62, and 0.58. Consistent with the bifurcation analysis, in the first and last of these cases, the model solution orbit approaches, respectively, a low [K^+^]_o_ (approximately 4 mM) and a high [K^+^]_o_ (approximately 35 mM) steady state asymptotically (black curves in Figures 4C1, C4, respectively), and, in the two intermediate cases, the solution orbit is oscillating and converges to a limit cycle (Figure 4C2, black curve) or spirals toward a steady state (Figure 4C3, black curve, corresponding to the dynamics of Figure 3B, bottom). The nullclines of $N_{K^{+},o}$ and $N_{Na^{+},n}$ are plotted as blue and red curves, respectively, in Figures 4C1–C4. The dynamics of $N_{Na^{+},n}$ are due to ion fluxes across the neuronal membrane, such that the corresponding nullcline is unaffected by the value of the glial sodium–potassium pump rate and, therefore, does not change between these plots. On the other hand, decreasing *f*_NaK_ has a marked effect on the nullcline of $N_{K^{+},o}$ . For *f*_NaK_ near 1, the two nullclines intersect for resting state values of [K^+^]_o_ and [Na^+^]_n_. By decreasing *f*_NaK_ at some value, the intersection point moves into the region where the red nullcline is increasing, approximately corresponding to the location of the Hopf bifurcation. Further decreasing *f*_NaK_ first, there is a regime in which the unstable state goes stable through a Hopf bifurcation (corresponding to the semi-elevated [K^+^]_o_ state and the orbit depicted in Figure 4C3), then it goes unstable again before the unstable state eventually vanishes (not visible in the figures), and the only remaining stable steady state is the elevated [K^+^]_o_ DB state.

### 3.3 Dependence on the NKCC1 cotransporter

**FIGURE 5:**
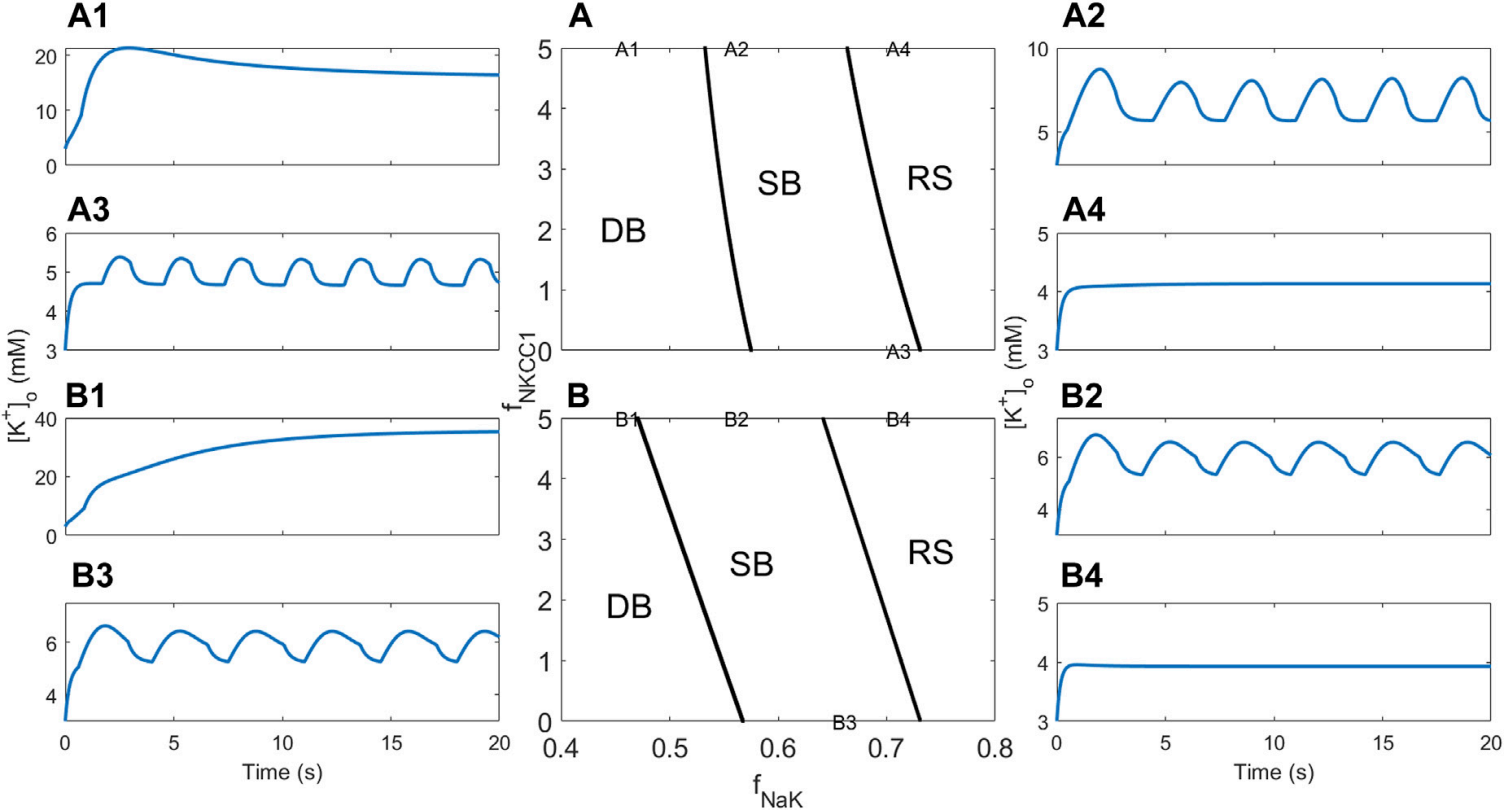
Bifurcation analysis and [K^+^]_o_ dynamics showing the effect of pump rate and NKCC1. **(A)** Two-parameter bifurcation diagram showing how dynamics of the RM1 depends on *f*_NaK_ and *f*_NKCC1_. The three regions indicated by RS, SD, and DB correspond to regions of the (*f*_NaK_, *f*_NKCC1_)–plane where the resting state, spontaneous discharge, and depolarisation block behaviour are observed, respectively. “**A1**”**–**“**A4**” in the figure indicate the locations in the parameter plane associated with simulations whose results are shown in **(A1–A4)**. **(B)** Same as **(A)** for RM2. “**B1**”**–**“**B4**” in the figure indicate the locations in the parameter plane associated with simulations whose results are shown in **(B1–B4)**.

In order to assess how the sodium potassium chloride (NKCC1) cotransporter affects the neuron–glia system’s propensity to show bursting behaviour, a two-parameter bifurcation analysis was performed where the NKCC1 factor *f*_NKCC1_ (the factor multiplied by the NKCC1 ion flux *J*_NKCC1_) is the second bifurcation parameter in addition to *f*_NaK_. The two-parameter analysis starts with the one-parameter bifurcation diagrams for *f*_NaK_ and then keeps track of the location of the lower and upper Hopf bifurcations in Figures 4A2, B2 (The location of the lower Hopf bifurcation is used instead of the location of the heteroclinic bifurcation since XPPAUT has difficulties tracking the location of the latter). This results in two curves that approximately separate the (*f*_NaK_, *f*_NKCC1_)-plane into regions corresponding to RS, SB, and DB behaviour. Figures 5A, B display these regions for RM1 and RM2, respectively. The steepness of these curves suggests that the sensitivity of neuronal excitability to variation in the NKCC1 ion flux rate is much smaller than sensitivity to variation in *f*_NaK_.

For RM1, four simulations were subsequently performed in which parameter combinations were selected from each of the three regions; simulation A1 displays DB behaviour (Figure 5A1), simulations A2 and A3 display SB behaviour (Figures 5A2, A3), and simulation A4 displays RS behaviour (Figure 5A4), as expected from the associated location of the parameters in the (*f*_NaK_, *f*_NKCC1_)-plane in Figure 5A. Similarly for RM2, four simulations were subsequently performed in which parameter combinations were selected from each of the three regions; simulation B1 displays DB behaviour (Figure 5B1), simulations B2 and B3 display SB behaviour (Figures 5B2, B3), and simulation B4 displays RS behaviour (Figure 5B4), as expected from the associated location of the parameters in the (*f*_NaK_, *f*_NKCC1_)-plane in Figure 5B. The results for RM2 are almost identical to RM1; the only major difference is the DB value of [K^+^]_o_, which is much higher in RM2 than in RM1 (compare Figures 5A1, B1).

### 3.4 Dependence on [K^+^]_g_

**FIGURE 6:**
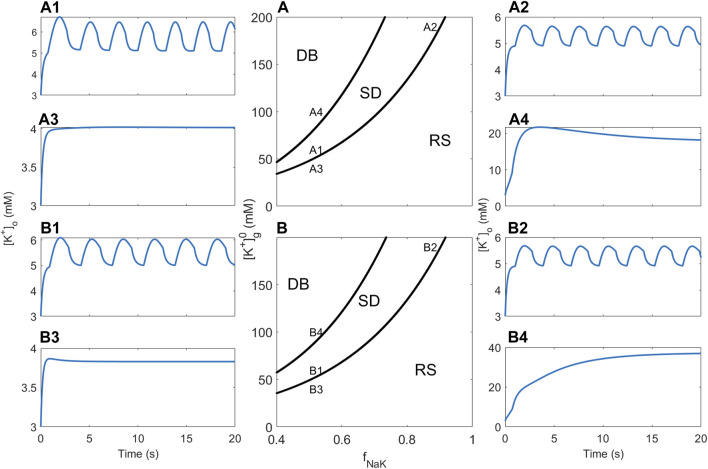
Bifurcation analysis and [K^+^]_o_ dynamics showing the effect of pump rate and [K^+^]_g_. **(A)** Two-parameter bifurcation diagram showing how dynamics of the RM1 depends on *f*_NaK_ and [K^+^]_g_. The three regions indicated by RS, SD, and DB correspond to regions of the $\left(f_{\text{NaK}},{\left[{\text{K}}^{+}\right]}_{\text{g}}\right)$ -plane where the resting state, spontaneous discharge, and depolarisation block behaviour, respectively, are observed. “**A1**”–“**A4**” in the figure indicate the locations in the parameter plane associated with simulations whose results are shown in **(A1–A4)**. **(B)** Same as **(A)** for RM2. “**B1**”**–**“**B4**” in the figure indicate the locations in the parameter plane associated with simulations whose results are shown in **(B1–B4)**.

Increased levels of K^+^ generally increase neuronal excitability. To investigate the models’ response to variable K^+^ total levels without changing the structure of the models, the initial glial K^+^ concentration $\left({\left[K^{+}\right]}_{g}\right)$ was selected as the quantity to represent total K^+^ levels. When [K^+^]_g_ is variable, the electroneutrality condition (4) may be disrupted such that the parameter *X*_g_ needs to be redefined in order to maintain glial electroneutrality. The aforementioned two-parameter bifurcation analysis was repeated with *f*_NKCC1_ replaced by [K^+^]_g_. Tracking the location of the lower and upper Hopf bifurcations for varying values of [K^+^]_g_, the curves in the (*f*_NaK_,[K^+^]_g_)-plane that approximately separate the parameter plane into three regions corresponding to RS, SB, and DB dynamics were obtained (Figures 6A, B corresponding to RM1 and RM2, respectively). Interestingly, in both diagrams, for [K^+^]_g_ below approximately 40 mM, the RS region extends to the zero pump rate, indicating that at low ${\left[K^{+}\right]}_{g}$ , bursting behaviour cannot be generated, regardless of the strength of the sodium–potassium pump. By contrast, the locations of both the lower and upper Hopf bifurcations increase with [K^+^]_g_ and the difference between them widens, indicating that the addition of K^+^ increases the propensity of the model to exhibit SB. Four simulations were subsequently performed in which parameter combinations were selected from each of the three regions: simulations A1 and A2 display SB behaviour (Figures 6A1, A2), A3 displays RS behaviour (Figure 6A3), and A4 displays DB behaviour (Figure 6A4), as expected from the associated location of the parameters in the (*f*_NaK_, [K^+^]_g_)-plane in Figure 6A. Similar to RM1, four simulations were performed in which parameter combinations were selected from each of the three regions: Simulations B1 and B2 and display SB behaviour (Figures 6B1, B2), simulation B3 displays RS behaviour (Figure 6B3), and simulation B4 displays DB behaviour (Figure 6B4). The main difference between RM1 and RM2 is that the DB value of [K^+^]_o_ is much higher in RM2 than in RM1 (compare Figures 6A4, B4).

## 4 Discussion

The present study demonstrates how various actors in the glial membrane can potentially influence neuronal excitability. This is achieved by developing two highly simplified mathematical models that describe ion concentration dynamics in the neuron–ECS–glia system. Central to the derivation of the reduced models from the full neuron–glia model of Øyehaug et al. (2012) is the replacement of action potential-induced neuronal currents by expressions that depend on neuronal Na^+^ and ECS K^+^ concentrations. The dynamics of the reduced models mimics that of the full model, and bifurcation analyses show that the dynamical repertoire of these models is similar to that of the full model and to previously published models (e.g., Cressman et al., 2009; Barreto and Cressman, 2011) and consistent with experimental observations (McBain, 1994; Jensen and Yaari, 1997; Feng and Durand, 2006; Ziburkus et al., 2006).

**FIGURE 7:**
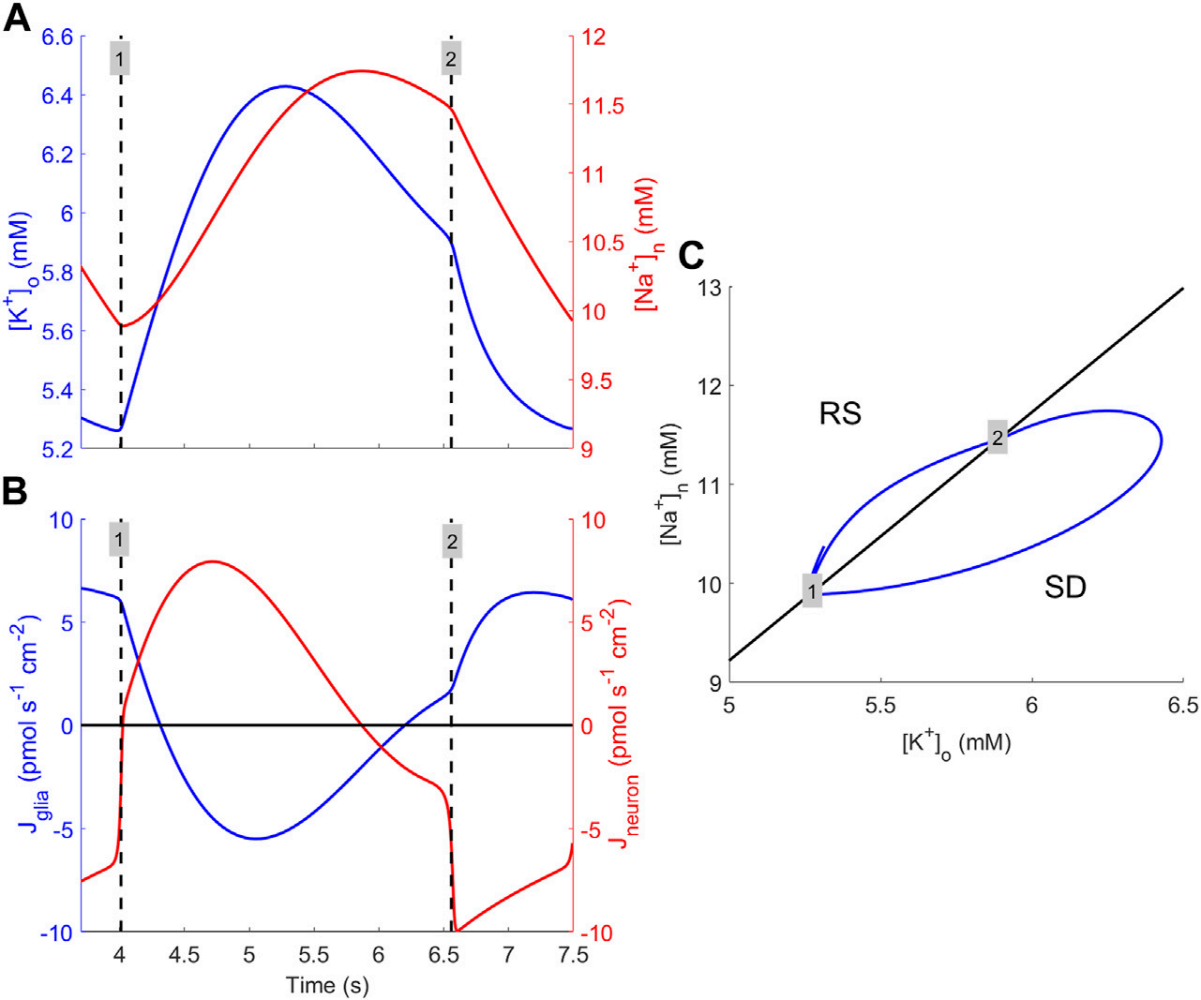
Explanation for how burst cycles are maintained. **(A)** [K^+^]_o_ (blue) and [Na^+^]_n_ (red) dynamics during one cycle of bursting obtained by numerically solving the RM2 model Eqs 10a, 10b using the default parameter set except the sodium–potassium pump rate, which is multiplied by 0.70 to generate periodic bursting solutions. The times of entering and exiting from the SD region are indicated (black dashed vertical lines) and labelled “1” and “2,” respectively. **(B)** Magnitude of glial transmembrane K^+^ flux (blue) and of neuronal transmembrane K^+^ and Na^+^ fluxes (red) during one cycle. The black vertical dashed lines and the numbering have the same meaning as in **(A)**. **(C)** Solution orbit during one cycle (blue) in the $\left({\left[K^{+}\right]}_{o},{\left[Na^{+}\right]}_{n}\right)$ -plane and the line that separates the RS and SD regions (black). Labels “1” and “2” refer to the times indicated in **(A)** and **(B)**, respectively.

The results of the bifurcation analyses provide evidence that the mechanism responsible for bursting is a Hopf bifurcation. Mathematically, this is clearly correct, but since the concept of a bifurcation is difficult to interpret directly into the physiological context, a clarification of the circumstances that enable periodic bursting is required. The bursting phenomenon is primarily caused by interaction between [K^+^]_o_ and [Na^+^]_n_, suggesting that RM2 is an appropriate venue for explaining how bursting behaviour is maintained. For solutions of RM2, where the selected parameter values are consistent with bursting, one cycle of the solution is considered (Figures 7A–C). At the time labelled “1,” the solution orbit enters the SD region (Figure 7C), which triggers a rapid switch in the neuronal ion flux from negative to positive. This causes [K^+^]_o_ and [Na^+^]_n_ to abruptly go from declining to increasing (Figures 7A, B). Once the solution orbit has entered the SD region, for a while, transmembrane sodium and potassium currents increase in magnitude such that [K^+^]_o_ and [Na^+^]_n_ increase. However, rising levels of [Na^+^]_n_ cause a decrease in the K^+^ neuronal efflux, which, in turn, leads to reduced [K^+^]_o_ and, ultimately, the solution orbit exits from the SD region and re-enters the RS region (Figure 7, “2”). In this region, both K^+^ and Na^+^ fluxes are substantially reduced compared to in SD, causing a decrease in both concentrations. This lasts until [Na^+^]_n_ becomes sufficiently small to allow the neuronal efflux of K^+^ to increase, permitting the orbit to re-enter the SD region (Figure 7, “1”), causing the initiation of another bursting episode.

The bifurcation analysis shows that the reduced models possess interesting properties, including the coexistence of two modes (*bistability*) and even three modes (*tristability*) of dynamics. Theoretical explorations into the dynamics of the mathematically tractable RM2 should be made to gain an improved understanding of this model and, if possible, pinpoint the mechanisms in the model that are responsible for the bistability and tristability phenomena. One specific question that should be addressed in this respect is why the model ends up in one of the stable states rather than the others, as seen in the simulation (Figure 3B, bottom), where, in the parameter regime of tristability, the model converges to the semi-elevated K^+^ state. The long-time behaviour of the model orbit depends on the location of the orbit in state space, i.e., which *basin of attraction* the initial state belongs to. Determining the boundaries between the various basins of attraction is non-trivial in complex high-dimensional systems but should be feasible in simpler models, such as RM1 and RM2, and thus represents a possible topic for future investigation. The aforementioned efforts will most likely reveal whether or not bi and tristability are artefacts of the choice of expressions in the model or of the simplifying assumptions that lead to the creation of the reduced models. Furthermore, similar investigations into the full model could be made to examine its potential for displaying tristability. If it has such a potential, one could argue that the coexistence of three types of dynamical behaviour could be of biological significance.

Without modifying the present models, in future studies, the significance of the *neuronal* sodium–potassium pump and neuronal sodium and potassium channels can be assessed using the methods employed in the present study. Furthermore, applying slight modifications and extensions of the models, future prospects for applying the reduced models to assess the roles of more glial actors and processes include, but are not limited to, the sodium-bicarbonate cotransporter NBC, the inwardly rectifying K^+^ (Kir) 4.1 channel (Sibille et al., 2015), the potassium–chloride cotransporter KCC1, and calcium-dependent channels. It is known that ECS and glia volumes are variable even under quite normal circumstances (Østby et al., 2009; Ullah et al., 2015). The impact of ECS and glia volume variation on model dynamics can be assessed using RM1. However, since RM1 and RM2 dynamics are qualitatively similar despite constant volumes in RM2 (compare Figures 4A2, B2), altering the parameters that govern volume change in RM1 will most likely not substantially affect the dynamics of this model.

Epilepsy has been linked to weakened handling of ECS K^+^ levels (Fröhlich et al., 2008a), astroglia dysfunction (Binder and Steinhäuser, 2006), and reduced overall activity of the sodium–potassium pump (Grisar et al., 1992), consistent with the increased propensity of the models of the present study to exhibit oscillatory ion concentration dynamics when glial uptake mechanisms are impaired. In the elevated [K^+^]_o_ model of epilepsy, local perturbations in ECS K^+^ levels spontaneously trigger neurons, which, in turn, lead to epileptic seizure activity (e.g., Bazhenov et al., 2004; Fröhlich et al., 2008a; Fröhlich et al., 2008b; Florence et al., 2012), which may be propagated throughout the brain tissue (Lebovitz, 1996; Park and Durand, 2006; Naze et al., 2015; Chizhov and Sanin, 2020). To describe mathematical conditions for the spreading of neuronal activity and for the termination of seizures, a spatial model is required where neuron–glia systems interact with other neuron–glia systems through the diffusion of ions and other agents or the propagation of electrical properties, i.e., neuron–ECS–glia network models (Park and Durand, 2006; Durand et al., 2010; Ullah and Schiff, 2010; Oschmann et al., 2018; Manninen et al., 2023). The simplicity of the reduced models makes them suitable as nodes in complex network models, in particular models that describe the spread of seizure activity. One specific question to be addressed by such models is how high [K^+^]_o_-induced neuronal activity (i.e., seizures) may spread in a multicellular neuron–glia network and how activity propagation and termination depend on parameters of individual neuron–ECS–glia systems and on properties of the interaction between these systems.

Potentially, network models may also be applied to describe *spreading depression* (Ayata and Lauritzen, 2015), which is characterised by a wave of intense but transient regional depolarisation of neurons and glia, and associated with high [K^+^]_o_ due to overload of ECS K^+^ clearance mechanisms. When neuronal activity is non-uniform across the neuron–ECS–glia system, spatial models on a cellular or sub-cellular spatial scale are required to describe phenomena such as *spatial K* ^*+*^ *buffering* (Orkand et al., 1966; Halnes et al., 2013; Witthoft et al., 2013), where glia absorb excess K^+^ at glial sites facing the synapse and release it at distant locations. A theoretical investigation of spatial potassium buffering and its putative role in ECS K^+^ clearance and the promotion or suppression of associated phenomena, such as SB and DB behaviour, represents an interesting avenue for future research.

Blood supply and thus the provision of oxygen and nutrients to neural tissue increase with enhanced neural activity, suggesting the existence of a complex interplay between neurons, glia, epithelial cells of the blood-brain-barrier, and non-cellular elements such as the ECS and the extracellular matrix, the elements of the so-called neurovascular unit (Attwell et al., 2010; Iadecola, 2017). In addition to its role as the most prominent signalling ion in the neuron-ECS-glia system, K^+^ contributes to signalling across the neurovascular unit (Paulson and Newman, 1987; Farr and David, 2011). A theoretical investigation of the intricate interaction between ion concentrations in the neuron-ECS-glia subsystem and blood flow regulation could combine the reduced models of the present study with models that account for migration of K^+^ ions through glia from perisynaptic to perivascular regions where K^+^ and other agents are assumed to contribute to dilation and constriction of blood vessels (as e.g. Farr and David, 2011; Witthoft et al., 2013). This kind of approach, where no subsystems are considered as isolated entities, but rather as interacting components, is very much aligned with the brain active milieu research initiative (Semyanov and Verkhratsky, 2021; 2022).

Reduced models, although expected to be less realistic than the more detailed full neuron–glia model, nevertheless reproduce qualitatively essential features of the full model. In addition, owing to the low complexity and relatively small number of tunable parameters, mechanisms responsible for certain phenomena are easily identified using simple models, demonstrated in this study in which analyses show that bursting behaviour in the neuron–glia system can be attributed to variability in the glial sodium–potassium pump rate, the NKCC1 cotransporter uptake rate, and glial K^+^ levels.

## Data Availability

The original contributions presented in the study are included in the article/Supplementary Material; further inquiries can be directed to the corresponding author.
