# Supplementary material for: Slow ion concentration oscillations and multiple states in neuron–glia interaction—insights gained from reduced mathematical models
Source: Front Netw Physiol. 2023 May 22;3:1189118. doi: 10.3389/fnetp.2023.1189118 (PMC10241345; doi:10.3389/fnetp.2023.1189118)
Supplement: Supplementary file 1 [file Presentation1.PDF]

Supplementary Material

1 SUPPLEMENTARY DATA

1.1 The glia model of the full model

The glia model equations of (Øyehaug et al., 2012) are

$$\frac{dN_{\mathrm{Na^+,g}}}{dt} = 10^{-2}\left(J_{\mathrm{Na}}^{(\mathrm{g})} - 3J_{\mathrm{NaKATPase}}^{(\mathrm{g})} + J_{\mathrm{NKCC1}} + J_{\mathrm{NBC}}\right) , \tag{S1a}$$

$$\frac{dN_{\mathrm{Na^+,o}}}{dt} = 10^{-2}\left(J_{\mathrm{Na}}^{(\mathrm{n})} + 3J_{\mathrm{NaKATPase,n}}\right) - \frac{dN_{\mathrm{Na^+,g}}}{dt} , \tag{S1b}$$

$$\frac{dN_{\mathrm{K^+,g}}}{dt} = 10^{-2}\left(J_{\mathrm{K}}^{(\mathrm{g})} + 2J_{\mathrm{NaKATPase}}^{(\mathrm{g})} + J_{\mathrm{NKCC1}}\right) , \tag{S1c}$$

$$\frac{dN_{\mathrm{K^+,o}}}{dt} = -\frac{dN_{\mathrm{Na^+,g}}}{dt} - \frac{dN_{\mathrm{Na^+,o}}}{dt} - \frac{dN_{\mathrm{K^+,g}}}{dt} , \tag{S1d}$$

$$\frac{dN_{\mathrm{HCO_3^-,g}}}{dt} = 10^{-2} \times 2J_{\mathrm{NBC}} , \tag{S1e}$$

$$\frac{dN_{\mathrm{HCO_3^-,o}}}{dt} = -\frac{dN_{\mathrm{HCO_3^-,g}}}{dt} , \tag{S1f}$$

$$\frac{dw_{\mathrm{g}}}{dt} = 10\, L_p \left(\Pi_{\mathrm{g}} - \Pi_{\mathrm{o}}\right) . \tag{S1g}$$

In order to arrive at the glia model equations in Eqs (2a – 2d) in the main document, the following actions are performed:

1. NBC is neglected. Thereby Eqs (S1e) and (S1f) can be omitted and the term J_{NBC} set to zero.
2. The derivatives on the right side of Eq (S1d) are written out and the corresponding ODE is placed first.
3. Eq (S1b) is replaced by an ODE for $N_{\mathrm{Na^+,o}}$ using ion number conservation $N_{\mathrm{Na^+,n}} = N_{\mathrm{Na^+,tot}} - N_{\mathrm{Na^+,o}} - N_{\mathrm{Na^+,o}}$ such that $dN_{\mathrm{Na^+,n}}/dt = -dN_{\mathrm{Na^+,o}}/dt - dN_{\mathrm{Na^+,o}}/dt$.
4. Having performed actions 1 – 3 it is observed that $dN_{\mathrm{K^+,o}}/dt + dN_{\mathrm{K^+,g}}/dt - dN_{\mathrm{Na^+,n}}/dt = 0$, i.e. $N_{\mathrm{K^+,o}} + N_{\mathrm{K^+,g}} - N_{\mathrm{Na^+,n}}$ is constant and the ODE for $N_{\mathrm{K^+,g}}$ is omitted.

The resulting model equations are

$$\frac{dN_{\mathrm{K^+,o}}}{dt} = 10^{-2}\left[J_{\mathrm{Na}}^{(\mathrm{n})} + 3J_{\mathrm{NaKATPase}}^{(\mathrm{n})} - \left(J_{\mathrm{K}}^{(\mathrm{g})} + 2J_{\mathrm{NaKATPase}}^{(\mathrm{g})} + J_{\mathrm{NKCC1}}\right)\right], \tag{S2a}$$

$$\frac{dN_{\mathrm{Na^+,n}}}{dt} = 10^{-2}\left[J_{\mathrm{Na}}^{(\mathrm{n})} + 3J_{\mathrm{NaKATPase}}^{(\mathrm{n})}\right], \tag{S2b}$$

$$\frac{dN_{\mathrm{Na^+,g}}}{dt} = 10^{-2}\left[J_{\mathrm{Na}}^{(\mathrm{g})} - 3J_{\mathrm{NaKATPase}}^{(\mathrm{g})} + J_{\mathrm{NKCC1}}\right], \tag{S2c}$$

$$\frac{dw_{\mathrm{g}}}{dt} = 10\, L_p \left[\Pi_{\mathrm{g}} - \Pi_{\mathrm{o}}\right], \tag{S2d}$$

consistent with the glia model equations in the main document.

1.2 Conversion factors

To demonstrate the need for conversion factors and quantify the magnitude of these factors, consider the ODE for ion numbers of an ion S of the form

$$\frac{dN_S}{dt} = \Phi_S J_S,$$

where $N_S = w[S]$ is the ion number variable, w is the ratio of cell volume to cell membrane area (with unit $[w] = \mu$m), [S] is the ion concentration with unit mmol/liter, Φ_S is the conversion factor and J_S is the ion flux. The unit of the derivative of N_S is

$$\mu\mathrm{m\,mmol/liter/ms} = 10^{-9}\mathrm{mol\,m/liter} = \frac{10^{-10}\,\mathrm{mol}}{\mathrm{cm}^2\,\mathrm{ms}}.$$

Considering the case that $J_S = g_S(V_m - E_S)/F$ (with units $[V_m - E_S] = m$V, $[g_S] = \mu\mathrm{S\,cm}^{-2} = \mu\Omega^{-1}\mathrm{cm}^{-2}$ and $[F]$ = C/mol) the flux J_S has unit

$$[J_S] = \frac{\mu\Omega^{-1}\,\mathrm{cm}^{-2}\mathrm{mV}}{\mathrm{Cmol}^{-1}} = 10^{-9}\frac{\Omega^{-1}\,\mathrm{Vmol}}{\mathrm{C\,cm}^2} = 10^{-9}\frac{\mathrm{mol}}{\mathrm{cm}^2\,\mathrm{s}} = \frac{10^{-12}\,\mathrm{mol}}{\mathrm{cm}^2\,\mathrm{ms}}.$$

Thus, matching units in the ODE is achieved by multiplying the right side by $\Phi_S = 10^{-2}$.

Similarly, consider the ODE for w;

$$\frac{dw}{dt} = \Phi_w L_p(\Pi_i - \Pi_o)$$

where $\Pi_i - \Pi_o$, the difference between extra- and intracellular osmolarities, has unit m mol/liter, such that the unit of $L_p(\Pi_i - \Pi_o)$ is

$$[L_p][\Pi_i - \Pi_o] = \mathrm{cm\,s}^{-1}\mathrm{mM}^{-1}\,\mathrm{mM} = \frac{\mathrm{cm}}{\mathrm{s}} = \frac{10^{-2}\,\mathrm{m}}{10^3\,\mathrm{ms}} = \frac{10^{-5}\,\mathrm{m}}{\mathrm{ms}}.$$

The unit of the left side is

$$[\frac{dw}{dt}] = \frac{\mu\,\mathrm{m}}{\mathrm{ms}} = \frac{10^{-6}\,\mathrm{m}}{\mathrm{ms}},$$

such that matching units is achieved by multiplying the right side by $\Phi_w = 10$.

1.3 Proof of constant volumes in RM2

In the baseline state, volumes are assumed to be in equilibrium, i.e. $dw_g/dt = 0$. Due to assumption that the sum of $[K^+]_o$ and $[Na^+]_o$ is constant, dw_g/dt remains zero for $t > 0$, as shown in the following. First, observe that since the net flux of total K^+ and Na^+ across the neuronal membrane is zero, the total number of K^+ and Na^+ ions within the neuron is constant. Since it is assumed that ion numbers are conserved across all compartments, this means that the total quantity of potassium and sodium in ECS and glia

$N^{(\mathrm{o+g})}_{\mathrm{K^+,Na^+}} = N^{\mathrm{o}}_{\mathrm{K^+}} + N^{\mathrm{g}}_{\mathrm{K^+}} + N^{\mathrm{o}}_{\mathrm{Na^+}} + N^{\mathrm{g}}_{\mathrm{Na^+}}$ is also conserved. Then,

$$\begin{aligned}
\frac{dw_{\mathrm{g}}}{dt} &= 10L_p\left[\Pi_{\mathrm{g}} - \Pi_{\mathrm{o}}\right] \\
&= 10L_p\frac{2}{w_{\mathrm{g}}}\left[w_{\mathrm{g}}([\mathrm{Na^+}]_{\mathrm{g}} + [\mathrm{K^+}]_{\mathrm{g}}) + \frac{1-\rho}{2}X_{\mathrm{g}} - w_{\mathrm{g}}([\mathrm{K^+}]_{\mathrm{o}} + [\mathrm{Na^+}]_{\mathrm{o}})\right] \\
&= 10L_p\frac{2}{w_{\mathrm{g}}}\left[N^{(\mathrm{o+g})}_{\mathrm{K^+,Na^+}} + \frac{1-\rho}{2}X_{\mathrm{g}} - (w_{\mathrm{o}} + w_{\mathrm{g}})\left([\mathrm{K^+}]_{\mathrm{o}} + [\mathrm{Na^+}]_{\mathrm{o}}\right)\right].
\end{aligned}$$

The terms within the brackets are constants, that is, since the expression is initially zero it must remain so for all $t > 0$. Thus $dw_{\mathrm{g}}/dt = 0$, i.e. glia and ECS volumes are constant.

Variable/parameter	Value	Description
$V_{\mathrm{m}}^{(\mathrm{n})}$	-70 mV	Neuronal membrane potential
$m_{\mathrm{Na,T}}$	0.0050*	Activation gate of the transient sodium current
$h_{\mathrm{Na,T}}$	0.9961*	Inactivation gate of the transient sodium current
$m_{\mathrm{Na,P}}$	0.0129*	Activation gate of the persistent sodium current
$h_{\mathrm{Na,P}}$	0.9718*	Inactivation gate of the persistent sodium current
n	0.0012*	Activation gate of the delayed rectifier potassium current
$m_{\mathrm{K,A}}$	0.1193*	Activation gate of the transient potassium current
$h_{\mathrm{K,A}}$	0.1205*	Inactivation gate of the transient potassium current
$V_{\mathrm{m}}^{(\mathrm{g})}$	-85 mV	Glial membrane potential
$[\mathrm{K}^+]_\mathrm{n}/[\mathrm{K}^+]_\mathrm{o}/[\mathrm{K}^+]_\mathrm{g}$	130/3/100 mM	Neuronal/ECS/glial potassium concentration
$[\mathrm{Na}^+]_\mathrm{n}/[\mathrm{Na}^+]_\mathrm{o}/[\mathrm{Na}^+]_\mathrm{g}$	10/146/15 mM	Neuronal/ECS/glial sodium concentration
$[\mathrm{Cl}^-]_\mathrm{n}/[\mathrm{Cl}^-]_\mathrm{o}/[\mathrm{Cl}^-]_\mathrm{g}$	50/134/5.00* mM	Neuronal/ECS/glial chloride concentration
$w_\mathrm{n}/w_\mathrm{o}/w_\mathrm{g}$	0.05/0.04/0.08 μm	Neuronal/ECS/glia volume to A ratio
$g_{\mathrm{leak,K}}$	66.06* μS cm^{-2}	Potassium leakage channel conductance
$g_{\mathrm{leak,Na}}$	20 μS cm^{-2}	Sodium leakage channel conductance
$g_{\mathrm{leak,f}}$	10 μS cm^{-2}	Unspecified leakage channel conductance
$E_{\mathrm{f,n}}$	-70 mV	Unspecified leakage channel reversal potential
$g_{\mathrm{Na,T}}$	5000 μS cm^{-2}	Transient sodium channel conductance
$g_{\mathrm{Na,P}}$	150 μS cm^{-2}	Persistent sodium channel conductance
$g_{\mathrm{K,DR}}$	5000 μS cm^{-2}	Delayed rectifier potassium channel conductance
$g_{\mathrm{K,A}}$	1000 μS cm^{-2}	Activating potassium channel conductance
$I_{\mathrm{NaKATPase,n,max}}$	2127* nA cm^{-2}	Maximum neuronal sodium-potassium pump rate
g_{K}	1696* μS cm^{-2}	Glial potassium channel conductance
g_{Na}	100 μS cm^{-2}	Glial sodium channel conductance
g_{Cl}	100 μS cm^{-2}	Glial chloride channel conductance
g_{NKCC1}	2 μS cm^{-2}	Glial cotransporter NKCC1 permeability
$J_{\mathrm{NaKATPase,g,max}}$	0.0921* nA cm^{-2}	Maximum glial sodium-potassium pump rate
$K_{\mathrm{m,Na}}$	8 mM	Sodium threshold of neuronal/glial pump rate function
$K_{\mathrm{m,K}}$	1 mM	Potassium threshold of neuronal/glial pump rate function
L_p	2×10^{-8} cm s^{-1} mM^{-1}	Glial membrane water permeability
X_g	13.52* μmol cm^{-2}	Number of impermeable ions in glia divided by A
ρ	0.5965*	Average charge of impermeable ions relative to e

Table S1. Full model variables with baseline values and parameters with values and descriptions. Neuronal parameter values (between second and third empty rows) are adopted from (Kager et al., 2000) and references to glial parameter values (below third empty row, $K_{\mathrm{m,Na}}$ and $K_{\mathrm{m,K}}$ are both neuronal and glial parameters) can be found in (Østby et al., 2009). Variable and parameter values indicated by an asterisk were derived from the model equations to ensure that the model is in a steady state at baseline. A and e are the glial surface area and the elementary charge, respectively.

Current density or rate	Expression	Description
$I_{\mathrm{leak,Na}}$	$g_{\mathrm{leak,Na}}\left(V_{\mathrm{m}}^{(\mathrm{n})}-E_{\mathrm{Na}}^{(\mathrm{n})}\right)$	Sodium leak current density
$I_{\mathrm{leak,K}}$	$g_{\mathrm{leak,K}}\left(V_{\mathrm{m}}^{(\mathrm{n})}-E_{\mathrm{K}}^{(\mathrm{n})}\right)$	Potassium leak current density
$I_{\mathrm{leak,f}}$	$g_{\mathrm{leak,f}}\left(V_{\mathrm{m}}^{(\mathrm{n})}-E_{\mathrm{f}}^{(\mathrm{n})}\right)$	Unspecified leak current density
$I_{\mathrm{Na,T}}$	$g_{\mathrm{Na,T}}m_{\mathrm{Na,T}}^{3}h_{\mathrm{Na,T}}\left(V_{\mathrm{m}}^{(\mathrm{n})}-E_{\mathrm{Na}}^{(\mathrm{n})}\right)$	Sodium transient current density
$I_{\mathrm{Na,P}}$	$g_{\mathrm{Na,P}}m_{\mathrm{Na,P}}^{2}h_{\mathrm{Na,P}}\left(V_{\mathrm{m}}^{(\mathrm{n})}-E_{\mathrm{Na}}^{(\mathrm{n})}\right)$	Sodium persistent current density
$I_{\mathrm{K,DR}}$	$g_{\mathrm{K,DR}}n_{\mathrm{K,DR}}^{2}\left(V_{\mathrm{m}}^{(\mathrm{n})}-E_{\mathrm{K}}^{(\mathrm{n})}\right)$	Potassium delayed rectifier current density
$I_{\mathrm{K,A}}$	$g_{\mathrm{K,A}}m_{\mathrm{K,A}}^{2}h_{\mathrm{K,A}}\left(V_{\mathrm{m}}^{(\mathrm{n})}-E_{\mathrm{K}}^{(\mathrm{n})}\right),$	Potassium transient current density
$I_{\mathrm{NaKATPase,n}}$	$I_{\mathrm{NaKATPase,n,max}}\frac{[\mathrm{Na}^+]_{\mathrm{n}}^{1.5}}{[\mathrm{Na}^+]_{\mathrm{n}}^{1.5}+K_{\mathrm{m,Na}}^{1.5}}\frac{[\mathrm{K}^+]_{\mathrm{o}}}{[\mathrm{K}^+]_{\mathrm{o}}+K_{\mathrm{m,K}}}$	Sodium-potassium pump current density
$\alpha_{m_{\mathrm{Na,T}}}$	$0.32\frac{-v-51.9}{\exp[-(0.25v+12.975)]-1}$	Forward rate for $m_{\mathrm{Na,T}}$
$\beta_{m_{\mathrm{Na,T}}}$	$0.28\frac{v+24.89}{\exp[0.2v+4.978)]-1}$	Backward rate for $m_{\mathrm{Na,T}}$
$\alpha_{h_{\mathrm{Na,T}}}$	$0.128\exp[-(0.056v+2.94)]$	Forward rate for $h_{\mathrm{Na,T}}$
$\beta_{h_{\mathrm{Na,T}}}$	$\frac{4}{\exp[-(0.2v+6)]+1}$	Backward rate for $h_{\mathrm{Na,T}}$
$\alpha_{m_{\mathrm{Na,P}}}$	$\frac{1}{\tau_{\mathrm{activation}}}\frac{1}{\exp[-(0.143v+5.67)]+1}$	Forward rate for $m_{\mathrm{Na,P}}$
$\beta_{m_{\mathrm{Na,P}}}$	$\frac{1}{\tau_{\mathrm{activation}}}\frac{\exp[-(0.143v+5.67)]}{\exp[-(0.143v+5.67)]+1}$	Backward rate for $m_{\mathrm{Na,P}}$
$\alpha_{h_{\mathrm{Na,P}}}$	$5.12\times10^{-8}\exp[-(0.056v+2.94)]$	Forward rate for $h_{\mathrm{Na,P}}$
$\beta_{h_{\mathrm{Na,P}}}$	$\frac{1.6\times10^{-6}}{\exp[-(0.2v+8)]+1}$	Backward rate for $h_{\mathrm{Na,P}}$
$\alpha_{n_{\mathrm{K,DR}}}$	$0.016\frac{-v-34.9}{\exp[-(0.2v+6.98)]-1}$	Forward rate for $n_{\mathrm{K,DR}}$
$\beta_{n_{\mathrm{K,DR}}}$	$0.25\exp[-(0.025v+1.25)]$	Backward rate for $n_{\mathrm{K,DR}}$
$\alpha_{m_{\mathrm{K,A}}}$	$0.02\frac{-v-56.9}{\exp[-(0.1v+5.69)]-1}$	Forward rate for $m_{\mathrm{K,A}}$
$\beta_{m_{\mathrm{K,A}}}$	$0.0175\frac{v+29.9}{\exp[(0.1v+2.99)]-1}$	Backward rate for $m_{\mathrm{K,A}}$
$\alpha_{h_{\mathrm{K,A}}}$	$0.016\exp[-(0.056v+4.61)]$	Forward rate for $h_{\mathrm{K,A}}$
$\beta_{h_{\mathrm{K,A}}}$	$\frac{0.5}{\exp[-(0.2v+11.98)]+1}$	Backward rate for $h_{\mathrm{K,A}}$

Table S2. Current densities and forward and backward rates describing the channels in the neuron model. The neuronal Nernst potential of ion species S is $E_{\mathrm{S}}^{(\mathrm{n})}=((RT/F)/z_{\mathrm{S}})\ln([\mathrm{S}]_{\mathrm{o}}/[\mathrm{S}]_{\mathrm{n}})$, z_{S} is the valence of S, R is Boltzmann's constant, T is the temperature in Kelvin and F is Faraday's constant. The forward and backward rates are given as function of the neuron membrane potential, here denoted v, adopted from Kager et al. (2000). For simplicity, v and other quantities with unit mV or mV^{-1} as well as forward and backward rates (with unit ms^{-1}) are given without units in the table. The time constant $\tau_{\mathrm{activation}}$ is set to 6 ms.

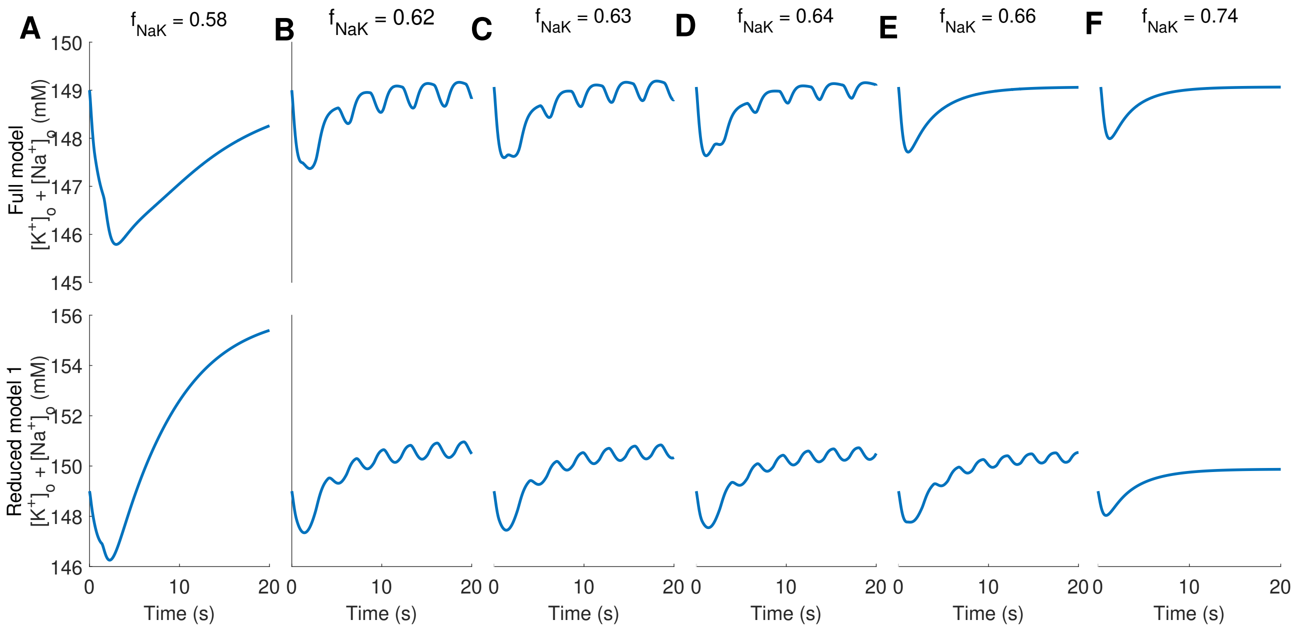

Figure S1. Comparison of the dynamics of $[K^+]_o + [Na^+]_o$ obtained in simulations using the full model (top) and RM1 (bottom). Columns (A) – (F) display dynamics for increasing values of the sodium-potassium pump rate obtained by multiplication by a factor f_{NaK} in the range 0.58 – 0.74, indicated at the top of each column.

REFERENCES

Kager, H., Wadman, W. J., and Somjen, G. G. (2000). Simulated seizures and spreading depression in a neuron model incorporating interstitial space and ion concentrations. *J. Neurophysiol.* 84, 495–512

Østby, I., Øyehaug, L., Einevoll, G. T., Nagelhus, E. A., Plahte, E., Zeuthen, T., et al. (2009). Astrocytic mechanisms explaining neural-activity-induced shrinkage of extraneuronal space. *PLoS Comp. Biol.* 5, e1000272. doi:10.1371/journal.pcbi.1000272

Øyehaug, L., Østby, I., Lloyd, C. M., Omholt, S. W., and Einevoll, G. T. (2012). Dependence of spontaneous neuronal firing and depolarisation block on astroglial membrane transport mechanisms. *J Comput. Neurosci.* 32, 147–165. doi:10.1007/s10827-011-0345-9
